# Mental health, well-being and support interventions for UK ambulance services staff: an evidence map, 2000 to 2020

**DOI:** 10.29045/14784726.2021.3.5.4.25

**Published:** 2021-03-01

**Authors:** Lucy V. Clark, Roberta Fida, Jane Skinner, Jamie Murdoch, Nigel Rees, Julia Williams, Theresa Foster, Kristy Sanderson

**Affiliations:** University of East Anglia; University of East Anglia; University of East Anglia; University of East Anglia; Welsh Ambulance Services NHS Trust; Swansea University; University of Hertfordshire; East of England Ambulance Service NHS Trust; University of East Anglia

**Keywords:** ambulance staff, mental health, well-being

## Abstract

**Background:**

Prior to COVID-19 there had been a renewed policy focus in the National Health Service on the health and well-being of the healthcare workforce, with the ambulance sector identified as a priority area. This focus is more important than ever as the sector deals with the acute and longer-term consequences of a pandemic.

**Aim:**

To systematically identify, summarise and map the evidence regarding mental health, well-being and support interventions for United Kingdom ambulance services staff and to identify evidence gaps.

**Method:**

Evidence mapping methodology of published and grey original research published in English from 1 January 2000 to 23 May 2020 describing the health risk, mental health and/or well-being of UK ambulance services staff including retired staff, volunteers and students. MEDLINE, EMBASE, PsychINFO, CINAHL and AMED databases, plus EThOS, Zetoc, OpenGrey and Google, were searched, alongside hand-searching of grey literature and bibliographies. Information was extracted on study aims, sample, design and methodology, funding source, country and key findings. Included studies were categorised into seven *a priori* theme areas.

**Results:**

Of 1862 identified articles, 45 peer-reviewed studies are included as well as 24 grey literature documents. Peer-reviewed research was largely observational and focused on prevalence studies, post-traumatic stress disorder or organisational and individual social factors related to health and well-being. Most grey literature reported the development and testing of interventions. Across all study types, underpinning theory was often not cited.

**Conclusion:**

To date, intervention research has largely been funded by charities and published in the grey literature. Few studies were identified on self-harm, bullying, sleep and fatigue or alcohol and substance use. Theoretically informed intervention development and testing, including adaptation of innovations from other countries and 24-hour workforces, is needed. This evidence map provides important context for planning of staff well-being provision and research as the sector responds to and recovers from the pandemic.

**PROSPERO registration number:**

CRD42018104659.

## Introduction

There are approximately 47,000 paid ambulance services staff in the United Kingdom (UK) (data from cqc.org.uk; scottishambulance.com; ambulance.wales.nhs.uk) and many more who have retired. These paid staff work alongside approximately 11,000 ambulance services volunteers, known as community first responders (CFRs), and thousands of unpaid students. For all these workers, whether working as clinicians, taking calls, transporting patients or in any other role, exposure to traumatic events and other workplace stressors is an integral part of their role. The COVID-19 pandemic has seen unprecedented impacts on healthcare delivery, including in the ambulance sector, and the National Health Service (NHS) is preparing for long-term impacts on staff well-being (Wallbank, 2020).

We know from international research that ambulance services’ staff face specific challenges and unique circumstances, and this working environment can lead to poor health and well-being including symptoms of fatigue, post-traumatic stress and depression (Petrie et al., 2018a; Varker et al., 2017). Support interventions, policies and procedures are essential to keep staff healthy, happy and in their jobs (Health Education England, 2019; Petrie et al., 2018b).

The health and well-being of ambulance services staff in the UK is a pressing issue; there continues to be both a shortage of paramedics nationally and a high attrition rate (Health Education England, 2019). As of 2017, according to NHS England, up to a third of paramedic posts were vacant in some parts of England (Public Health England, 2017). These staff shortages are due to staff leaving ambulance services, partly due to increasing opportunities for work in other healthcare settings, but also likely partly due to the pressure of work in a service in which demand continues to increase (Public Health England, 2017). Attempts have been made to address these issues for paramedics in Scotland through legislation such as the Health and Care (Staffing) (Scotland) Act 2019, and in other professions through the Nurse Staffing Levels (Wales) Act 2016, both of which recognise the importance of staff well-being and present strategies that support healthcare staff in delivering their roles.

In 2017, Health Education England (HEE) announced a new Commission on the mental well-being of NHS staff in their draft Health and Care Workforce Strategy for England to 2027 (Public Health England, 2017). The final report, written to support the NHS Long Term Plan, was published in February 2019, and sets the challenge of 32 recommendations with the aim of improving staff mental well-being (Health Education England, 2019), with one of these recommendations to address mental well-being challenges within the paramedic workforce. The Welsh Ambulance Service NHS Trust (WAST) have been particularly proactive; in 2018, in a submission to the Welsh Assembly *Government Inquiry into Suicide Prevention*, they acknowledged that the mental well-being of ambulance service staff could not be overlooked, and stated that their organisation was committed to developing accessible support services for colleagues (Welsh Ambulance Services NHS Trust, 2018).

The future of the UK healthcare system relies on its staff, with the health and well-being of staff a priority action area for the NHS People Plan (NHS England, 2019). However, a better understanding of the health risks, mental health and well-being of ambulance services staff is required to generate the testing of interventions targeted at their specific risks. It is not enough to rely on international evidence (Sofianopoulos et al., 2012; Stanley et al., 2016; Varker et al., 2017), as the UK ambulance services have a distinct set of challenges. This includes having to work to strict response times in a climate with increasing demands and an efficiency drive, turnaround times at hospital being delayed by a lack of hospital beds and ongoing changes to the scope of the roles (Eaton et al., 2018; Wankhade, 2016).

## Evidence mapping

Evidence maps are a relatively new method for identifying, organising and summarising scientific evidence on a broad topic (Bragge et al., 2011; Miake-Lye et al., 2016; Schmucker et al., 2013). While the traditional systematic review, meta-analysis, scoping review and rapid review are all methods used to synthesise data, evidence maps serve a different purpose. They aim to answer a broader question than systematic reviews – ‘what evidence exists in a particular area?’ (Callahan et al., 2012; Tricco et al., 2018) – and to ensure new research is informed by the existing evidence. This is a robust method of reviewing literature when there is a need to collate and summarise studies to look for gaps in knowledge, rather than to provide synthesis or aggregate data like in systematic reviews (Moher et al., 2010) or to provide a descriptive narrative of the results like in scoping reviews (Arksey & O’Malley, 2005). The capacity for breadth in evidence mapping allows the identification of evidence gaps in order to guide future research efforts. Evidence maps are based on an explicit but broad research question in relation to the field of enquiry, informed by researchers or research funding bodies who can identify gaps in the evidence, which in turn will create opportunities for new research. The search for, and collection of, appropriate studies uses explicit and reproducible methods at each stage (Arksey & O’Malley, 2005; Katz et al., 2003), with results presented as a systematic detailed description (through a map) of the literature defined by the review question (Gough et al., 2012, 2017).

Collating and summarising the evidence base (over the past 20 years) related to the health risks, mental health, well-being and support interventions in UK ambulance services staff is an essential first step in obtaining an overview of the breadth of research activities in this area, and this methodology is most appropriate in this instance (Miake-Lye et al., 2016). The aims of this review are to systematically identify, organise and summarise the evidence and identify evidence gaps, in order to guide future research questions and methodology and enable stakeholders to make evidence-based decisions on health and well-being policies and procedures. This evidence map will provide important context for planning of staff well-being provision and research as the sector responds to, and recovers from, the pandemic.

## Methods

To demonstrate methodological rigour and transparency, and ensure its relevance for decision making, we referred to the PRISMA reporting guidelines for scoping reviews, which also apply to evidence mapping methods (PRISMA-ScR) (Tricco et al., 2018), and used the PRISMA flowchart (Moher et al., 2009).

### Review protocol

The study protocol was registered on the PROSPERO international register of systematic reviews, registration number 42018104659 (https://www.crd.york.ac.uk/PROSPERO/display_record.php?RecordID=104659).

### Search strategy

We conducted searches in MEDLINE, PsychInfo, CINAHL, AMED and EMBASE (final search run on 23 May 2020) to identify studies investigating the health and well-being of UK ambulance services staff. We also undertook searches of grey literature using a number of overlapping approaches (Adams et al., 2016) including databases (e.g. OpenGrey, TRIPdatabase, Zetoc and EThOS), websites and personal contact with relevant organisations (e.g. charities with an interest in the mental health of emergency services staff) and a popular internet search engine (Google.com) using the terms ‘ambulance’, ‘personnel’ or ‘worker’ and ‘staff health’, and limited to UK websites – checking the first 50 results (Dewa et al., 2016). In order to capture both current and past literature, and potentially view any changing trends in research focus in this area, the search period was extended to just over 20 years (1 January 2000 to 23 May 2020). The search terms were determined in consultation with experts in the area of the health and well-being of UK ambulance services staff. The terms used for searching the published literature are detailed in Table 1. Publication citations were exported from electronic search interfaces to EndNote (X8).

**Table 1.**
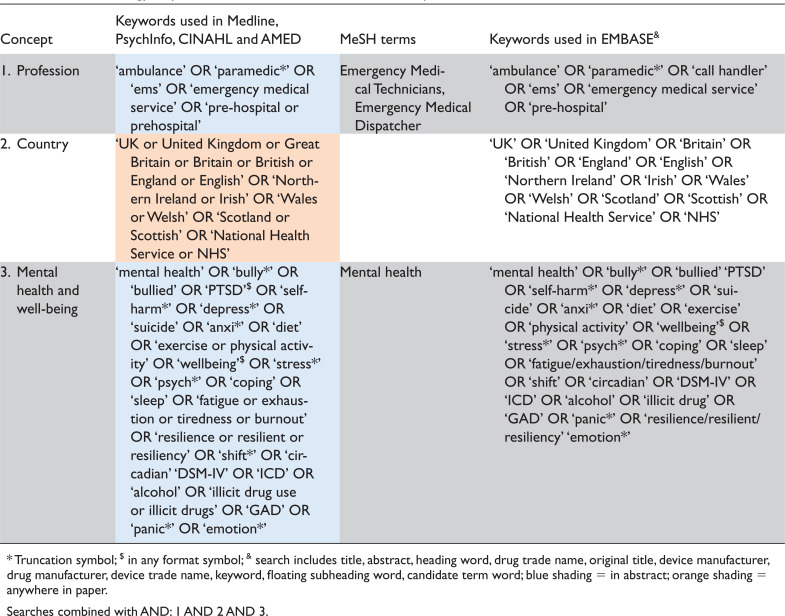
Search strategy: Keywords and MeSH terms for evidence map review.

## Inclusion and exclusion criteria

Studies were eligible for inclusion if the population of interest was UK (England, Scotland, Wales and Northern Ireland) ambulance services staff at any grade and in any role, including volunteer community first responders, paramedic students and retired staff and volunteers. The focus of the study had to be health risk, mental health and well-being and/or exploration of associated influencing factors, and the study had to present original research (e.g. it was not solely an opinion piece, any type of review or an editorial or commentary), to have been published since 2000 in a peer-reviewed journal or as a report that was publicly available, or be an available thesis, and to have been published in English.

Studies were excluded if the area of health related solely to ambulance services staff interactions with the public (e.g. empathy levels) rather than health risks, mental health and/or well-being. Studies were also excluded if they solely investigated aspects of staff professional skills and training (e.g. student preparedness based on the quality of their clinical placement).

A single study can have multiple publications, and as such, all relevant papers for each study were presented with clarification. This process prevented counting one study multiple times and misrepresenting the number of studies in a particular area. The final list of publications that met inclusion criteria was sent to four experts in the field to help identify any missing or ongoing research.

## Screening

References and documents were managed with the aid of reference management software EndNote (X8). A three-tier screening process was undertaken in which one reviewer (LVC) initially supressed duplicate references; the resulting list was then screened electronically by LVC for obviously irrelevant papers, by title and abstract. The full text of the remaining papers was screened against predetermined inclusion criteria; papers excluded at this stage were independently verified by a second reviewer (KS), with any disagreement resolved by discussion and consensus between the two reviewers.

### Data extraction and positioning the relevant evidence in the map (i.e. charting)

Information extracted from the studies included aims/objectives, study design, sample size and participants (including age, gender, setting and staff type (volunteer/paid/NHS), grade and role), data sources/methodology (qualitative/quantitative), funding source (UK RC, NIHR, stakeholders) and key findings (with any theory underpinning them). Authors were contacted for clarification when study information was unclear.

### Data synthesis

Studies that met the inclusion criteria were categorised according to the focus of the research, adapted from those described previously by Varker and colleagues (2017) as relevant to emergency services workforces in a similar evidence map conducted in Australia. By replicating this method, we promote international comparability and support this as a useful framework. The seven research foci (or domains) were:

1. Interventions related to health risks, mental health and/or well-being;
2. Potentially traumatic PTSD-related events;
3. Prevalence/incidence of health risks, mental health and/or well-being;
4. Psychological factors relating to work-related injury;
5. Sleep and fatigue;
6. Organisational factors relating to health risks, mental health and/or well-being;
7. Social/individual factors relating to health risks, mental health and/or well-being.

## Results

**Figure 1:**
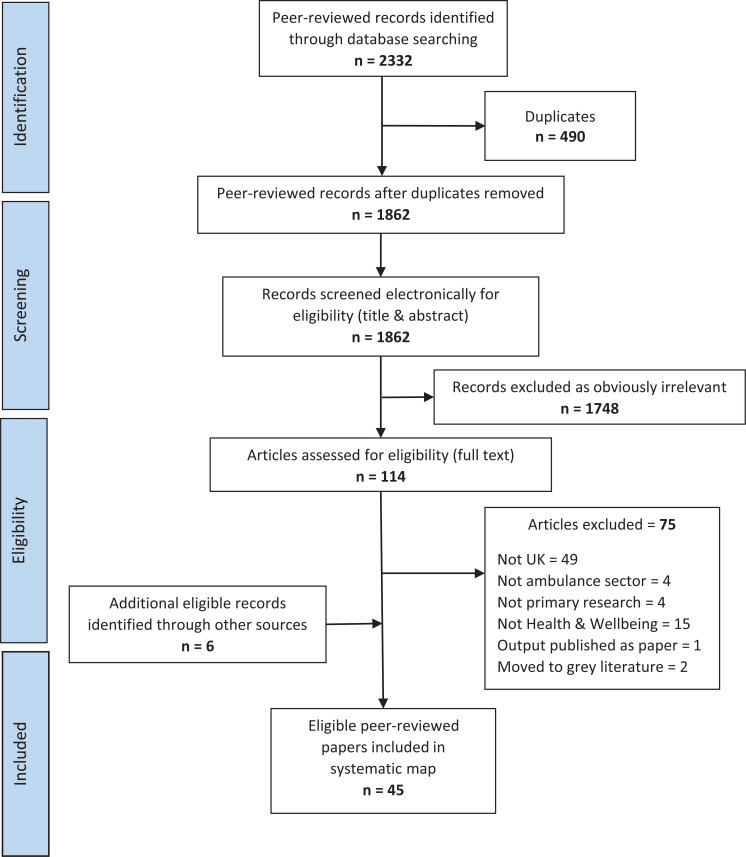
Figure 1. Preferred Reporting for Systematic Review and Meta-analysis (PRISMA) flow diagram for peer-reviewed paper screening.

The initial searches identified 2332 potentially relevant papers. Medline returned 514 papers; PsychInfo = 806, CINAHL = 303, AMED = 3, EMBASE = 706. After removing 470 duplicates, the resulting list included 1862 papers. The full text of 114 potentially relevant papers was then screened and an additional six relevant papers were found through searching reference lists. A total of 45 peer-reviewed papers were included in the final mapping process; a flowchart of the search is presented in Figure 1. Eighteen grey literature documents and six theses were identified from grey literature searches. An evidence map of recent research examining the health and well-being of UK ambulance services staff was produced, presented by type of evidence – peer-reviewed or grey (Tables 2 and 3). An overview of the characteristics and main outcomes of these studies is presented in the supplementary materials (Supplementary 1).

**Table 2.** Evidence map of number of peer-reviewed papers examining the health and well-being of UK ambulance services staff by research focus.

Research focus		Interventions	PTSD	Prevalence/incidence	Psychological factors of work-related injury	Sleep/fatigue	Organisational factors relating to health and well-being	Social and individual factors relating to health and well-being
Currently serving staff								
**1**	Aisling et al. 2016		✓	✓			✓	✓
**2**	Alexander and Klein 2001			✓			✓	✓
**3**	Barody 2016	✓						✓
**4 & 5**	*Bennett et al. 2004 & 2005		✓	✓			✓	✓
**6**	Clompus and Albarran 2016						✓	✓
**7**	Coxon et al. 2016						✓	✓
**8**	Davis et al. 2019		✓					
**9**	Drury et al. 2013						✓	✓
**10**	Forster 2020	✓					✓	
**11**	**Granter et al. 2019						✓	
**12**	Jenkin et al. 2007						✓	
**13**	Johnson et al. 2005			✓				
**14**	Kirby et al. 2016					✓	✓	
**15**	Machen et al. 2007						✓	
**16 & 17**	*Mahony 2001 & 2005						✓	
**18**	Mars et al. 2020						✓	✓
**19**	**McCann et al. 2013						✓	
**20**	Misra et al. 2009		✓					
**21**	Mutambudzi et al. 2020							✓
**22**	Nelson et al. 2020						✓	✓
**23**	Newbury-Birch et al. 2017						✓	
**24 & 25**	*O’Hara et al. 2014 & 2015						✓	
**26**	Power and Alison 2017						✓	✓
**27**	Price 2006						✓	
**28**	Rolfe et al. 2020							✓
**29**	Roy et al. 2020						✓	✓
**30**	Scott 2007							✓
**31 & 32**	*Shepherd and Wild 2014a & 2014b		✓					✓
**33**	Soh et al. 2016						✓	✓
**34**	Sprigg et al. 2007						✓	
**35**	Tehrani 2019	✓	✓					
**36**	Treglown et al. 2016							✓
**37**	Turnbull et al. 2017						✓	
**38**	Wild et al. 2016		✓					
Student paramedics								
**39**	Jennings 2017						✓	✓
**40**	Twinley 2012						✓	
**41 & 42**	*Williams 2013a & 2013b							✓
Volunteers								
**43**	Davies et al. 2008							✓
**44**	Kindness et al. 2014						✓	✓
**45**	Phung et al. 2018						✓	

*denotes two papers with same first author;

** denotes this is a paper from a study that has another paper with a different first author.

**Table 3.** Evidence map of number of grey literature publications examining the health and well-being of UK ambulance services staff by research focus.

	**Research focus**						
	Interventions	PTSD	Prevalence/incidence	Psychological factors of work-related injury	Sleep/fatigue	Organisational factors relating to health and well-being	Social and individual factors relating to health and well-being
Mind currently serving staff only								
** 1**	Huxley et al. 2018 – Mind evaluation	✓						
** 2**	Mind 2015a – Strand 1.1			✓				
** 3**	Mind 2016a – Strand 1.2	✓					✓	
** 4**	Mind 2016b – Strand 4	✓						
** 5**	Mind 2017a – New recruits, scoping						✓	
** 6**	Mind 2017b – Call handlers, scoping						✓	
** 7**	Mind 2018 – Call handlers, evaluation	✓						
** 8**	Mountford 2018 – Mind 1-year	✓					✓	✓
** 9**	New Economics Foundation 2017 – Mind	✓						
**10**	Robinson et al. 2016 – Mind Strand 5	✓					✓	
**11**	Rowe et al. 2019 – Mind						✓	✓
**12**	Wild 2016 – Mind Strand 3	✓						✓
**13**	Wild and Tyson 2017 – Mind	✓						✓
**14**	Wilson et al. 2016 - Mind Strand 2	✓					✓	✓
Mind currently serving staff, student paramedics, volunteers and retired staff								
**15**	Mind. 2015b – Scoping ambulance			✓			✓	
**16**	Mind 2016c – Suicide						✓	
**17**	Mind 2019a			✓			✓	
Theses and reports, currently serving staff								
**18**	Harper 2013							✓
**19**	Hicks Balgobin 2016	✓						✓
**20**	Leather 2016						✓	
**21**	Miller 2003	✓	✓					✓
**22**	Moreland 2006							✓
**23**	Richards 2011							✓
**24**	Rutter 2018		✓					✓

### Research focus: peer-reviewed work

As can be seen from the map in Table 2, there are 45 identified original peer-reviewed papers but only 39 original research projects, as six studies had two publications from the same research, indicated by an asterisk. Of the 39 research projects, nine compared ambulance services staff to at least one other group of health professionals, one reported emergency services staff as one group of emergency services staff, without reporting results separately, and the remaining 29 studies looked only at ambulance services staff including students, current employees and volunteers, in any role.

Organisational factors as they relate to health risks, mental health problems and/or well-being were the most commonly investigated area of research, reported in 27 studies. These include operational aspects such as exposure to traumatic incidents, undertaking shift work, potential risks due to work intensity, lack of resources/training or job demands and aspects related to the presence of resources within the workplace to support staff such as organisational and management structures and/or change, training and support, performance priorities, feelings of work satisfaction or perceived workplace support.

The research predominantly investigated the health risks, mental health and well-being of currently serving individual staff, sometimes specifically reporting outcomes for front line emergency staff such as paramedics. Three studies included student paramedics (Jennings, 2017; Twinley, 2012; Williams, 2013a & 2013b), three included control room staff, call handlers or dispatch staff (Coxon et al., 2016; Sprigg et al., 2007; Turnbull et al., 2017) and three included volunteer community first responders (CFR) (Davies et al., 2008; Kindness et al., 2014; Phung et al., 2018). Many studies included staff in different roles but did not always report results by role.

Seven studies, from as far back as 2004 and up to 2019, investigated post-traumatic stress disorder (PTSD) (Aisling et al., 2016; Bennett et al., 2004 & 2005; Davis et al., 2019; Misra et al., 2009; Shepherd and Wild 2014a & 2014b; Wild et al., 2016), although many more investigated job stressors including the impact of responding to particularly difficult jobs, and the impact of particular organisational structures or change. There were several areas of research where few studies were detected through the mapping process. These include the impact of shift work on sleep and fatigue (Kirby et al., 2016), factors associated with staff suicide (Mars et al., 2020), the prevalence and impact of abuse from members of the public (Newbury-Birch et al., 2017; Sprigg et al., 2007) and the effects of current performance indicators on staff mental health (Price, 2006).

Table 1 in Supplementary 1 shows the characteristics of the 39 studies. Seventeen studies used quantitative research methods, 17 used qualitative methods and four used mixed methods. Most were prevalence or observational studies, and just seven studies were longitudinal in design. Sixteen studies did not state if they had been funded, four stated they were not funded and the remaining studies were funded from a variety of sources including the NHS, the NIHR and Wellcome.

Although it is likely that all of the peer-reviewed literature studies are grounded in theory, just 14 reported clear theoretical frameworks to their work, with little overlap. These included theories of trauma, dissociation and intrusive memories (Bennett et al., 2004 & 2005) and various, mostly older, theories of work-related stress, emotional labour and professional identity (Barody, 2016; Clompus & Albarran, 2016; Coxon et al., 2016; Granter et al., 2019; Jennings, 2017; Johnson et al., 2005; Mahony, 2001 & 2005; McCann et al., 2013; Rolfe 2020; Treglown et al., 2016; Turnbull et al., 2017; Williams, 2013a & 2013b). Scott’s (2007) research on the expression of humour by emergency staff was influenced by observations of death and dying.

## Research focus: grey literature

As can be seen from the map in Table 3, we found 24 relevant documents in the grey literature, 17 of which were related to work funded by the charity Mind. We also found one unpublished research report and six unpublished theses.

Of the 24 documents, one compared ambulance services staff to another group of health professionals (Hicks Balgobin, 2016). Some of Mind’s work included staff from some or all of the emergency services (fire, police, search & rescue), and the data for ambulance services staff are not always reported separately. We chose to include documents even when this was the case, and have tried to extract the ambulance service-specific information where possible. Most documents looked at current paid staff in ambulance services, although Mind’s work also generally included volunteers, and they also report separately on the experiences of new recruits, call handlers and staff from Black, Asian and Minority Ethnic (BAME) groups. One thesis investigated students as they started work (Miller, 2003). Most of the theses reported on paramedics and/or emergency medical technicians.

Much of Mind’s work investigated interventions to understand and/or improve the health risks, mental health and well-being of ambulance staff, with a number of their other reports detailing scoping work undertaken in preparation for the intervention work. Interventions varied from those specifically designed to improve some aspect of health and well-being such as resilience (New Economics Foundation, 2017; Wild, 2016; Wild & Tyson, 2017), to interventions addressing mental health awareness, managing mental health at work (Wilson et al., 2016), training for line managers focusing on managing mental health in the workplace (Wilson et al., 2016) and interventions tailored to specific groups such as call handlers (Huxley et al., 2018) and those working in call centres (Mind, 2018). Mind has also undertaken more complex multi-pronged approaches to changing the culture in emergency services workplaces to try to get people to feel more able to talk about their mental health and to seek support (Mind, 2016a & 2016b). This includes the set-up and evaluation of a confidential information line (InfoLine) just for emergency services staff to get information on mental health, advice and signposting to local support services. It also includes the evaluation of the training and performance of Blue Light Peer Support set up by Mind (Robinson et al., 2016), and the effectiveness of the mental health networks, led by six local Mind networks across England (Mountford, 2018).

There were two theses investigating PTSD (Miller, 2003; Rutter, 2018), and another looking at the impact on staff of exposure to traumatic incidents (Harper, 2013). Organisational factors relating to health risks, mental health and well-being, the most commonly researched area in the peer-reviewed literature, were also a relatively common area of research in the grey literature, due to the scoping work, but often alongside the reporting of interventions, rather than alone.

As shown in Table 2 in Supplementary 1 of the 24 documents reported in the grey literature, nine used qualitative methodology, eight used quantitative methodology and seven used mixed methods.

Although it is likely that most, if not all, of the grey literature studies were underpinned by theory, theoretical frameworks were only identifiable from three documents, all of which were theses (Leather, 2016; Moreland, 2006; Richards, 2011).

## Discussion

Using evidence mapping methodology, we mapped the current evidence relating to the health risks, mental health and well-being of ambulance services staff and associated influencing factors, and of support interventions. Peer-reviewed research focused on prevalence studies and observational research on PTSD or organisational and individual/social factors and their relation to mental health and well-being. While more balanced, much of the grey literature documents described the development and testing of interventions. There were very few studies on post-traumatic stress disorder or staff suicide, and none on psychological factors of work-related injury. There were a few studies exploring the volunteer experience, and a few exploring the student experience, but none involving retired staff. Country-specific studies beyond England were limited to five studies in Northern Ireland, Scotland or Wales.

This evidence map is based on a comprehensive and systematic search, which included grey literature sources in an attempt to reduce any potential publication bias (Adams et al., 2016). Defining the boundaries of an evidence map is a somewhat subjective step, which could be considered a limitation, although to mitigate this we were guided by the ambulance services’ experts on our team. Evidence maps do not assess the quality of studies, or bias in their methodologies, and so it is not possible to make judgments about the quality of the research and evaluation presented. Similarly, we found a diversity of theoretical frameworks within a number of studies without a clear direction for designing interventions, indicating that further work is required to assess and synthesise different theories to inform the development and evaluation of interventions to improve well-being.

There were several areas where very few studies, if any, were detected through the mapping process, including suicide risk in staff. Given the evidence of a heightened risk of death by suicide among paramedics (Mars et al., 2020), the Association of Ambulance Chief Executives (AACE) has developed employee mental health guidance for ambulance services, which includes suicide prevention initiatives. Given the number of studies in the review, there was little research investigating the health and well-being of students and new recruits, who are known to be more vulnerable (McManamny et al., 2013), and none analysing data by length of tenure, which could be associated with resilience (Gayton & Lovell, 2012), or by operational versus non-operational roles which will likely influence support needs, not least because many non-operational roles do not require working at night.

There is a body of international research on the health and well-being of emergency services personnel (Petrie et al., 2018a; Sofianopoulos et al., 2012; Stanley et al., 2016; Varker et al., 2017), although few intervention studies (e.g. Jerome et al., 2020; McKeon et al., 2019), indicating that the shortage of published intervention studies in this group is not just a UK phenomenon. A review by Varker and colleagues (2017) reports a number of international studies looking at the effects of ambulance work, including shift work, on sleep and fatigue (Courtney et al., 2010, 2013; Paterson et al., 2014; Pyper & Paterson, 2016; Sofianopoulos et al., 2011). As sleep disturbance and fatigue not only affect the health of staff, but potentially compromise the role of front line staff by increasing the risk of accidents, injuries and errors, the impact of shift work on mental health including sleep and fatigue should be a future research priority.

The mental health charity Mind (http://www.mind.org.uk), through their Blue Light Programme, has made a significant contribution to understanding impacts on the health and well-being of emergency services personnel, including those working in ambulance services. It is their work which makes up most of the intervention testing. Their most recent 2019 report summarising the findings from their work demonstrates that since 2015: staff were more likely to say that their organisation encourages them to talk about mental health and supports people with mental health problems well; staff were more aware of support available to help them manage their mental health; excessive workload has continued to impact mental well-being, but trauma became more commonly reported; and PTSD rates have remained high (Mind, 2019b). Mind present recommendations for how emergency services and professional, research and government bodies can build on their findings.

There is also currently ongoing research in this space, including COVID-19 studies that discuss impacts on staff, such as the COVID-19 Ambulance Response Assessment (CARA) study being conducted by the College of Paramedics. Below are some examples of the research currently underway. Wild and colleagues (2018) are undertaking a randomised controlled trial testing internet-delivered cognitive training for resilience to prevent future PTSD in student paramedics, and an international longitudinal study (the International Paramedic Anxiety Wellbeing and Stress study) is exploring paramedic mental health on a global scale (Asbury et al., 2018). A national research study led by Yorkshire Ambulance Service and the University of Lincoln is investigating ambulance services staff well-being in a Health Education England (HEE) funded study (SWAP). We have identified further studies for which ambulance staff are eligible along with other NHS staff: Mindshine3 is investigating mindfulness interventions in NHS staff (ISRCTN15424185) and Ways Back to Work is assessing the feasibility of a case management-style intervention to facilitate a return to work in NHS staff off work sick with mental health disorders (ISRCTN14621901).

While it is known that ambulance staff are exposed to potentially stressful and traumatic situations, and the nature of their work (unpredictable finish times, long hours of driving, unpredictable breaks) increases the risk of chronic stress, not enough has been done to investigate strategies that could be used to prevent or manage occupational stress at both an organisational and individual/social level. Based upon the evidence in the UK to date, ambulance services are unlikely to meet the recommendations of the HEE report in addressing staff and learner well-being (Health Education England, 2019), and have an incomplete evidence base from which to derive a sector-led response.

There is little evidence on whether current actions to support staff well-being are working. This evidence map has described the pre-COVID-19 challenges and intervention options facing the ambulance sector workforce. It provides important context for future planning of staff well-being provision and research as the sector responds to and recovers from the pandemic. A combined organisational and individual approach to testing interventions for past, present and future ambulance staff mental health and well-being could potentially benefit more staff in the longer term and across multiple outcomes relevant to the individual and the NHS.

## Acknowledgements

The authors would like to thank Anna Parry, Fiona Bell, Kelly Hird and Sasha Johnston, who contributed to the literature search by reviewing the papers we identified and identifying additional papers and ongoing work. The authors also thank Matthew Smith, academic librarian in the Faculty of Medicine at the University of East Anglia.

## Author contributions

KS and LC conceived the study and devised the search strategy. All authors contributed to the design. LC conducted the searches and screened all titles, abstracts and full-text articles in consultation with KS. LC extracted the data in consultation with KS. LC and KS wrote the manuscript. RF, JS, JM, NR, JW and TF critically reviewed the protocol and manuscript for important intellectual content prior to submission. All authors approved the final submitted version. KS and LC act as joint guarantors for this article.

## Conflict of interest

JW is on the editorial board of the BPJ.

## Data sharing

All data in this evidence map are documented in the manuscript. No additional data are available.

## Ethics

Not required.

## Funding

None.
